# Biomechanical Dynamics of Cranial Sutures during Simulated Impulsive Loading

**DOI:** 10.1155/2015/596843

**Published:** 2015-04-06

**Authors:** Z. Q. Zhang, J. L. Yang

**Affiliations:** Institute of Solid Mechanics, Beijing University of Aeronautics and Astronautics, Beijing 100191, China

## Abstract

*Background*. Cranial sutures are deformable joints between the bones of the skull, bridged by collagen fibres. They function to hold the bones of the skull together while allowing for mechanical stress transmission and deformation. *Objective*. The aim of this study is to investigate how cranial suture morphology, suture material property, and the arrangement of sutural collagen fibres influence the dynamic responses of the suture and surrounding bone under impulsive loads. *Methods*. An idealized bone-suture-bone complex was analyzed using a two-dimensional finite element model. A uniform impulsive loading was applied to the complex. Outcome variables of von Mises stress and strain energy were evaluated to characterize the sutures' biomechanical behavior. *Results*. Parametric studies revealed that the suture strain energy and the patterns of Mises stress in both the suture and surrounding bone were strongly dependent on the suture morphologies. *Conclusions*. It was concluded that the higher order hierarchical suture morphology, lower suture elastic modulus, and the better collagen fiber orientation must benefit the stress attenuation and energy absorption.

## 1. Introduction

Cranial sutures are composite mechanical structures, which typically include two interdigitating components (the bones) and a thin more compliant interfacial layer (the collagen fibers) [1, 2]. The biocomposite structures thereby can function to bear and to transmit loads, to absorb energy, and to provide flexibility to accommodate growth and predatory protection [3–6].

In the bone-suture structures, geometrical morphology is a key determinant of mechanical and biological functions such as static load transmission, stiffness, strength, and energy absorption. The characterization of the geometry of various suture interfaces has been theoretically studied by Li and his coauthors [7, 8] along with a number of experimental numerical studies on these bone-suture systems [3, 9–12]. For example, cranial bone sutures were found to absorb more energy per unit volume during impact loading than monolithic cranial bone [3]. In addition, the bending strength of cranial bone sutures was found to increase with an increase in interdigitation [3, 11]. A numerical study of an interfacial crack with hierarchical sinusoidal suture found that the hierarchical wavy interface morphology can greatly enhance the ability of materials to resist interlaminar delamination and crack propagation [12]. However, a comprehensive and systematically quantitative understanding of the underlying role of geometry in the dynamical behavior and mechanisms is still lacking.

Meanwhile, finite element analysis (FEA) has been successful in developing a better understanding of the mechanical behavior of the sutures. Based on the FEA method, the influences of suture material properties (i.e., isotropic, transversely isotropic, and viscoelastic), suture morphologies, and direction of loading (i.e., perpendicular or parallel to the suture) on the mechanical behavior of the bone-suture structure were briefly reported by Rayfield [13], Jasinoski et al. [14], and Maloul et al. [15]. For example, Maloul et al. found that the suture mechanical behavior is impacted by morphologic factors (interdigitation and connectivity) that can alter their role in reducing total strain energy absorption.

It should be noted that almost all the previous studies only focused on the mechanical behavior of a single (first-order) bone-suture structure and did not consider the effects of hierarchical (higher-order) suture [16], although Jaslow and Biewener [4] pointed out the importance of the cranial suture with hierarchical-like morphology. In addition, the scope of these studies was limited to the mechanical behavior of bone-suture structure under static loading or cycling loading conditions from the viewpoint of strength and fracture toughness. In reality, however, dynamic responses of the bone-suture structure often occur. A typical functionality of the bone-suture structure is to protect various fragile organs inside it, such as brain, from external impact. Hence, a fast stress wave attenuation of the bone-suture structure under impulsive loading becomes vitally important. Building on this paper, here, we explore the role of hierarchical design on the underlying fundamental dynamics of bone-suture structure using FEA method.

In the present work, three two-dimensional FE models with different suture morphologies including straight, pure sinusoidal, and two-order hierarchical sinusoidal were developed. We aim to explore how morphological features (structural hierarchy) influence the dynamic responses of the suture and surrounding bone under impulsive loading. In addition, the effects of suture material properties (Young's modulus and collagen fibre orientation) on the dynamic responses of the suture and surrounding bone are examined.

## 2. Materials and Methods

**Figure 1:**
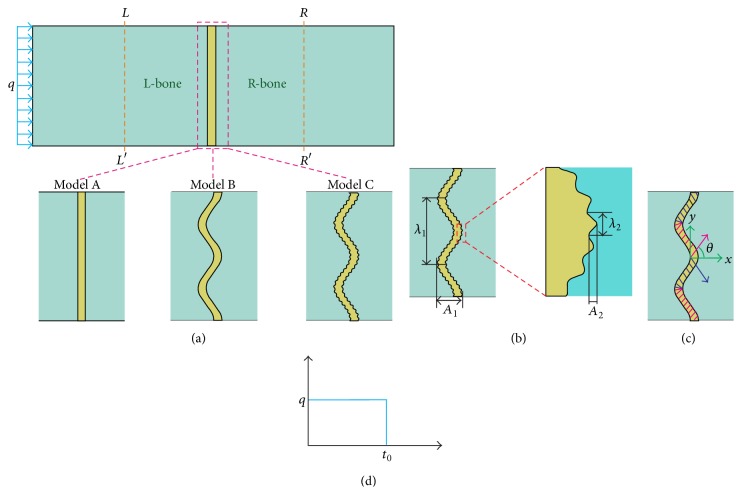
(a) Three different bone-suture models: model A with straight suture, model B with first-order sinusoidal suture, and model C with hierarchical sinusoidal suture. (b) Geometric parameters of model C. (c) Fiber arrangement inside the suture. (d) History of impulsive loading applied on the left side of the bone-suture model.

Idealized FE models of the bone-suture structure were developed in Abaqus/Explicit CAE (Simulia, USA) using two-dimensional FE model. The dimensions of the bone-suture complex were 12 mm × 4 mm, and the suture width *d* was approximately 0.25 mm. Based on the biological suture joints observed in the natural prototypes, the complicated suture morphology can be as simple as a sinusoid or exhibit a complex multiple wavelength pattern and/or a hierarchical fractal-like structure of shorter wavelengths superposed onto longer wavelengths [17, 18]. Thus, three wavy sutures were modeled with different morphologies: straight (model A), pure sinusoidal (model B), and two-order hierarchical sinusoidal (model C) to explore the role of hierarchical geometry in suture behaviors (Figure 1(a)). In model B, the suture structure was a sinusoidal wave of amplitude *A*_1_ = 0.25 mm and wavelength *λ*_1_ = 2 mm. In model C, the first-order suture structure was the same as that described in model B, and the second-order structure was a sinusoidal wave with amplitude *A*_2_ = 0.025 mm and wavelength *λ*_2_ = 0.15 mm (Figure 1(b)).

**Figure 2:**
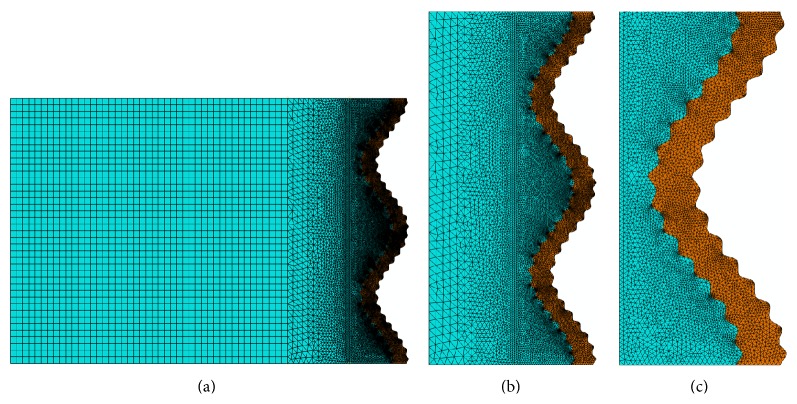
Typical element meshes at different length scales (half of model C).

Within Abaqus CAEPro, the three models were then meshed using 3-node plane stress linear triangular elements and 4-node bilinear plane stress quadrilateral elements. To obtain more reliable and convergent results, the minimum mesh size in the region of the hierarchical wavy sutures was set to be less than *d*/10. Therefore, the density of element mesh was significantly increased in the hierarchical wavy suture region (Figure 2). In addition, we considered that there were no gaps between the suture and bone meshes, and perfect adhesion was assumed.

In all FE models, the bone was treated as an isotropic material with Young's modulus *E*_*b*_ = 6000 MPa and Poisson's ratio *μ*_*b*_ = 0.27, based on an average of values used in many previous investigations (e.g., [19–21]). The suture was considered as an isotropic material to simulate a random orientation of collagen fibers and an orthotropic material to simulate the directional properties of the collagen matrix. In the isotropic suture models, Young's modulus (*E*_*s*_) of the sutural collagen fibers was assigned six values: 50, 100, 200, 300, 400, and 500 MPa, and Poisson's ratio (*μ*_*s*_) was set at 0.3 [14]. These choices of materials parameters were used to capture quantitatively the roles of suture in impact attenuation. In the orthotropic suture models, Young's modulus in the fiber and orthogonal directions were set at *E*_*s*1_ = 80 MPa and *E*_*s*2_ = 20 MPa, with corresponding values of Poisson's ratio of *μ*_*s*12_ = 0.4 and *μ*_*s*21_ = 0.1 [14]. The shear modulus was *G*_*s*_ = 20 MPa [14]. In order to explore the effects of fiber arrangement on the dynamic responses of the interdigitated suture, five orientations (*θ* = 15°, 30°, 45°, 60°, 75°) were simulated (Figure 1(c)). For all simulations, the densities of bone and suture were set at *ρ*_*b*_ = 2.06 g/cm^3^ and *ρ*_*s*_ = 1.06 g/cm^3^, provided by Wang et al. in previous studies [22].

A rectangular impulsive loading (*q*) of 50 kPa was applied to the left edge of the bone-suture complex (Figure 1(a)). The applied pulse length (*c*_0_ *t*_0_) was much less than the L-bone length, where *t*_0_  ( = 0.04 *μ*s) is the pulse duration and *c*_0_ is the elastic wave velocity of the bone (Figure 1(d)). To avoid the longitudinal edge effects, symmetrical boundary conditions were employed in the upper and lower edges of the complex. The total simulated time (*t*_end_) was set to be 35 *μ*s.

Overall, a total of 28 idealized FE models were analyzed. For each analysis, the von Mises stress at every point (element) in the bone-suture structure for all times was recorded. Based on these recorded data, the time histories of the average Mises stresses at section *L*-*L*′  (*σ*_*bL*_(*t*)) and at section *R*-*R*′  (*σ*_*bR*_(*t*)) were calculated to describe the dynamic responses of the L-bone and the R-bone, respectively (Figure 1(a)). A ratio between the first peak (*P*_*L*1_) and the second peak (*P*_*L*2_) of the stress history *σ*_*bL*_(*t*) was defined as

(1) $$\begin{array}{c} \eta =\frac{P_{L1}}{P_{L2}}. \end{array}$$

Similarly, the ratio (*δ*) between the first peak (*P*_*L*1_) of the stress history *σ*_*bL*_(*t*) and the peak stress (*P*_*R*1_) of the stress history *σ*_*bR*_(*t*) was obtained by

(2) $$\begin{array}{c} \delta =\frac{P_{L1}}{P_{R1}}. \end{array}$$

The ratios *η* and *δ* were used to evaluate the stress attenuation characteristics in the L-bone and R-bone.

In order to investigate the stress distribution of the L-bone under the impact load *q*, the stress uniformity index (SUI) was given by

(3) $$\begin{array}{c} \text{SUI}=\frac{\sqrt{\sum_{i=1}^{N}\left(\Delta S_{i}/S\right)\sigma_{i}^{2}\left(t\right)-{\left(\sum_{i=1}^{N}\left(\Delta S_{i}/S\right)\sigma_{i}^{}\right)}^{2}}}{q}, \end{array}$$

where *σ*_*i*_ represented Mises stress for L-bone element *i*, Δ*S*_*i*_ represented the area of the L-bone element *i*, *S* was the total area of the L-bone, and *N* was the total number of the L-bone elements.

In addition, the time history of the strain energy of the suture (SE) was measured. The mean strain energy (MSE) of the suture during the total time *t*_end_ could be computed:

(4) $$\begin{array}{c} \text{MSE}=\frac{1}{t_{\text{end}}}\overset{t_{\text{end}}}{\underset{0}{\int }}\text{SE}\left(t\right)\text{d}t. \end{array}$$

To study the effect of suture morphology on the strain energy, we introduced the MSE ratio (*e*) by

(5) $$\begin{array}{c} e=\frac{\text{MSE}}{{\text{MSE}}_{B}}, \end{array}$$

where MSE_*B*_ was the baseline mean strain energy. For the isotropic suture, the MSE of model A with *E*_*s*_ = 50 MPa was set as a baseline mean strain energy. For the orthotropic suture, the MSE of model B with *θ* = 15° was set as a baseline mean strain energy.

Parametric studies were performed to determine the effects of suture morphologies, Young's modulus *E*_*s*_, and fiber orientation *θ* on the impact attenuation, stress distributions, and the suture strain energy. *E*_*s*_ varied between 50 MPa and 500 MPa. *θ* varied from 15° to 75°.

The postprocessing calculations described in this section were performed in MATLAB.

## 3. Results

### 3.1. Attenuation of Dynamic Stress in L-Bone and R-Bone

**Figure 3:**
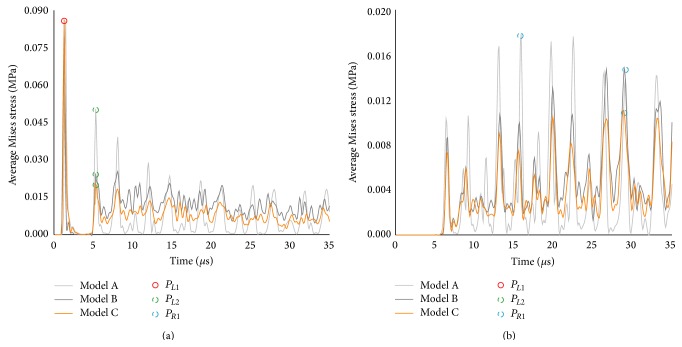
Time histories of the average Mises stresses at section *L*-*L*′ (a) and at section *R*-*R*′ (b).

For three different suture morphologies with *E*_*s*_ = 50 MPa, the magnitude of the transient sectional stresses *σ*_*bL*_(*t*) and *σ*_*bR*_(*t*) decreased with the number of hierarchies (Figure 3). The second peaks *P*_*L*2_ in model B and model C were all much lower than model A (Figure 3(a)). Comparing Figure 3(a) with Figure 3(b), it was found that the magnitude of the sectional stress *σ*_*bR*_(*t*) was much less than the sectional stress *σ*_*bL*_(*t*) for each model, suggesting that the suture had a good capability of impact attenuation.

**Figure 4:**
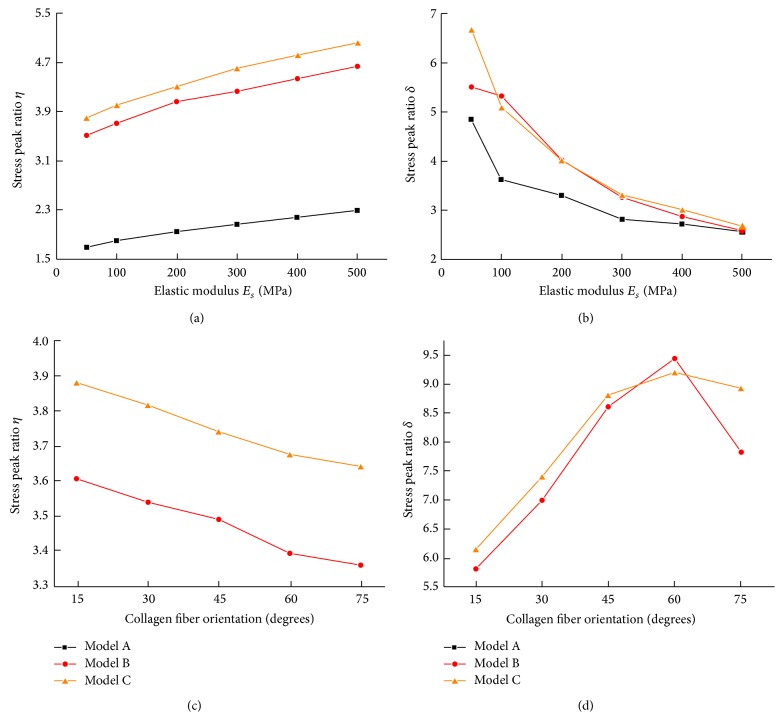
Variations of (a) stress peak ratios *η* and (b) *δ* in the isotropic models as a function of the suture elastic modulus. Variations of (c) stress peak ratios *η* and (d) *δ* in the orthotropic models as a function of the collagen fiber orientation.

Figure 4 showed the effects of Young's modulus and collagen fiber orientation on the sectional stress ratios *η* and *δ* for different suture morphologies. For a given suture morphology, the stress ratio *η* approximately linearly increased with increasing Young's modulus *E*_*s*_ (Figure 4(a)). By contrast, the stress ratio *δ* decreased rapidly with the increase of Young's modulus *E*_*s*_ (Figure 4(b)). In particular, the stress ratios of the three models were nearly equivalent at *E*_*s*_ = 500 MPa (Figure 4(b)). In the orthotropic models, the sectional stress ratio *η* decreased gradually when the collagen fiber orientation angle *θ* increased from 15° to 75° (Figure 4(c)). Moreover, there was a nonlinear relationship between the stress ratio *δ* and the collagen fiber orientation angle *θ*. As *θ* increased, *δ* increased until the transition angle (*θ* = 60°) was reached, at which point *δ* again decreased (Figure 4(d)).

### 3.2. Distribution of Dynamic Stress in L-Bone

**Figure 5:**
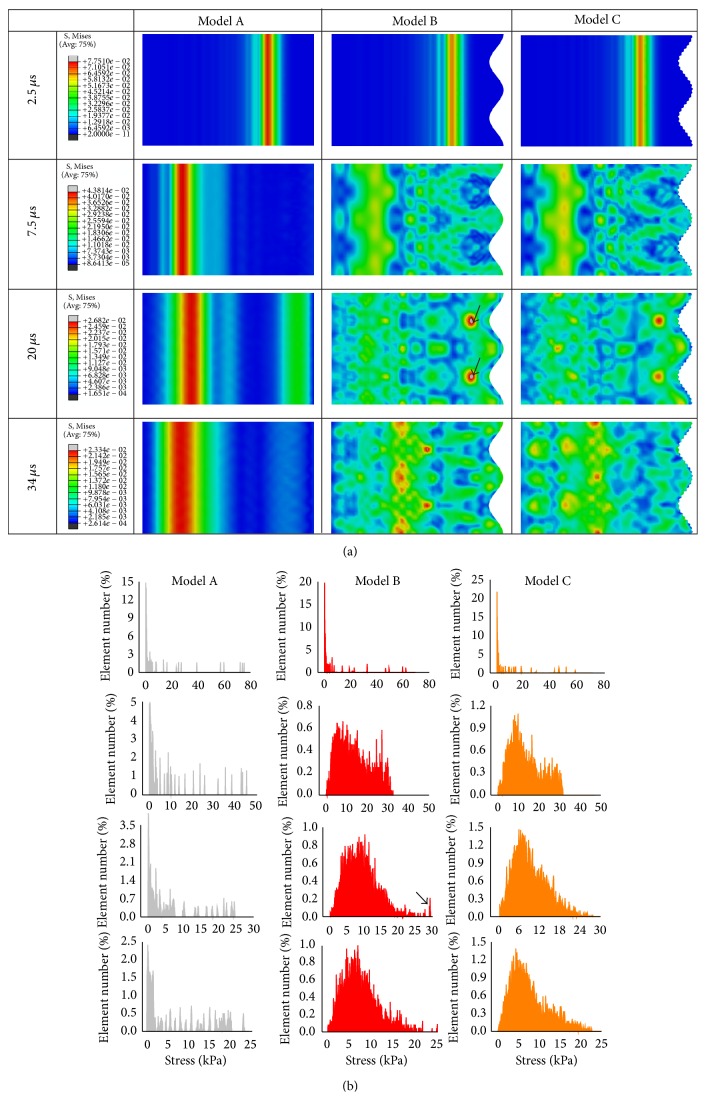
Distribution of von Mises stress in L-bone for different suture models: (a) contours of von Mises stress field and (b) the corresponding histograms.

In the isotropic models with *E*_*s*_ = 50 MPa, the stress distribution of the L-bone in the process of dynamic simulations was shown in Figure 5(a). The stress distribution showed great differences between the straight suture model (model A) and the interdigitated models (models B and C), suggesting that the dynamic stress distribution of the L-bone significantly depended on the suture morphologies. Correspondingly, the element Mises stresses of the L-bone were examined in all three models (Figure 5(b)). It depicted that the interdigitated models showed a greater concentration of stress values than that seen in the straight suture model, especially in the later simulations (i.e., *t* = 20 *μ*s, 34 *μ*s).

**Figure 6:**
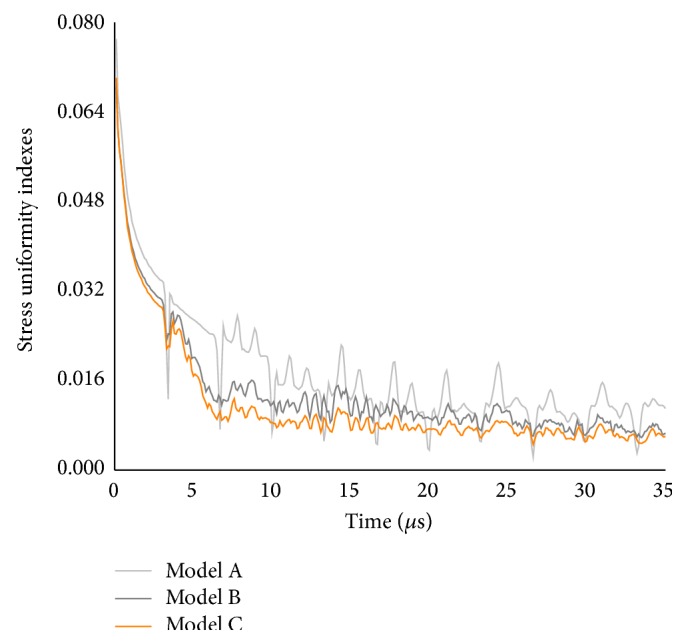
Stress uniformity index as a function of time for three isotropic suture models.

In order to gain a more fundamental understanding of the effect of suture morphology on the stress distribution within the L-bone, in Figure 6 we plotted the representative stress uniformity index (SUI) evolution of three isotropic suture models with *E*_*s*_ = 50 MPa. It could be seen from the figure that both the SUIs of model B and model C dropped sharply at first and then decreased slowly with the increase of simulation time, whereas the SUI of model A exhibited a sequence of oscillating behaviors varying with time. Furthermore, the hierarchical suture could greatly reduce the SUI of L-bone, comparing with the straight suture and the first-order suture (Figure 6).

**Figure 7:**
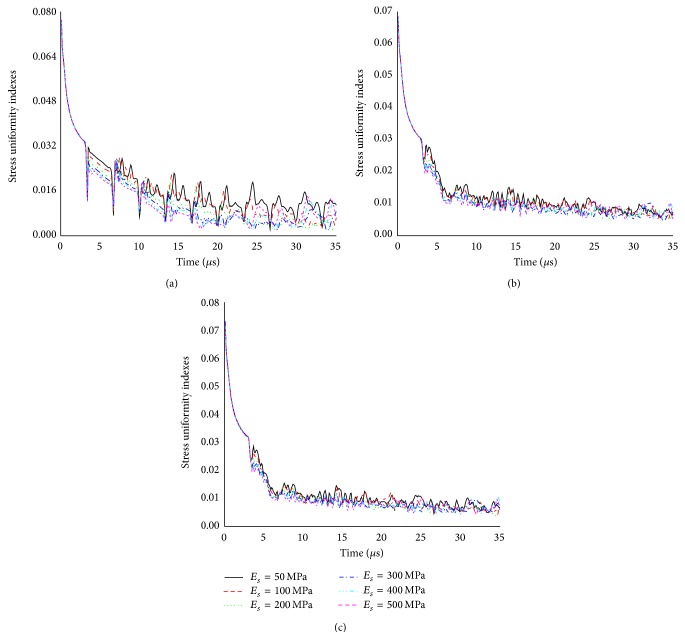
The influences of suture elastic modulus on the SUI of L-bone for three isotropic models: (a) model A, (b) model B, and (c) model C.

**Figure 8:**
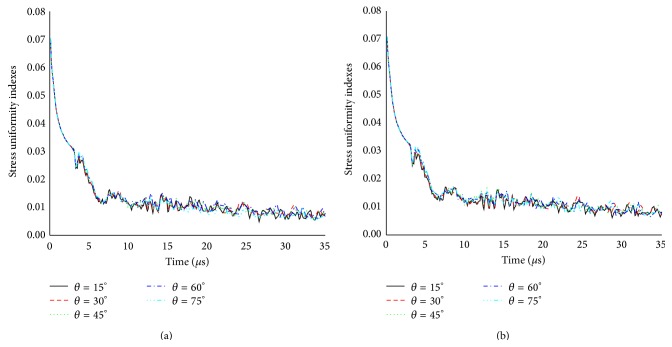
The influences of collagen fiber orientation on the SUI of L-bone for two orthotropic models: (a) model B and (b) model C.

The influences of suture elastic modulus and collagen fiber orientation on the SUI of L-bone for three models were summarized in Figures 7 and 8, respectively. For a given time, the larger suture elastic modulus *E*_*s*_ was, the smaller SUI was (Figure 7(a)). Such characteristics were also found in Figures 7(b) and 7(c), but they were not very noticeable. In the orthotropic models, the effect analysis of the collagen fiber orientation showed negligible qualitative differences in the SUI (Figure 8). These observations suggested that the suture morphology played a main role in the stress distribution within the L-bone.

### 3.3. Suture Strain Energy

**Figure 9:**
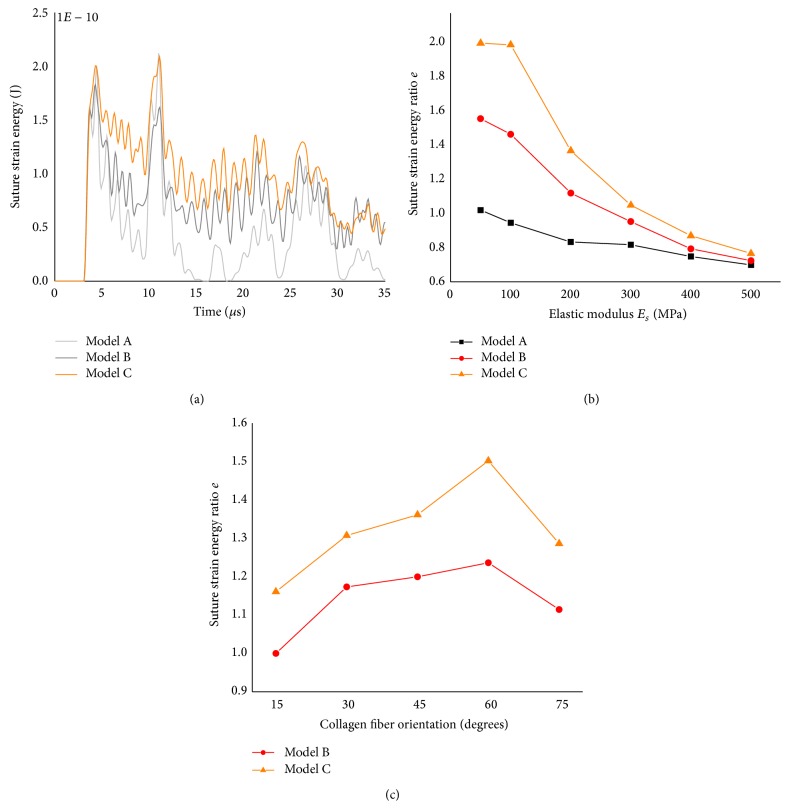
Suture strain energy for different suture models: (a) time history of the strain energy, (b) the influence of suture elastic modulus on the suture strain energy ratio, and (c) the influence of collagen fiber orientation on the suture strain energy ratio.

For the isotropic suture with *E*_*s*_ = 50 MPa, the strain energy-time histories of three different models were shown in Figure 9(a). It was clearly seen that the interdigitated suture could store more strain energy than the straight suture at a given time. The effects of suture elastic modulus and collagen fiber orientation on the strain energy were also investigated.  Figure 9(b) displayed the suture MSE ratio calculated by (5) as a function of the elastic modulus *E*_*s*_. Each line referred to a different suture morphology. The plot showed that the MSE ratio decreased when the suture became much stiffer.

In the orthotropic models, the variations of MSE ratio with the collagen fiber orientation *θ* for models B and C were represented in Figure 9(c), showing that the MSE ratio first increased and then decreased with increasing magnitude of collagen fiber angle. Therefore, there existed an optimal fiber orientation for maximum MSE ratio, indicating that the optimal fiber arrangement should store more strain energy.

## 4. Discussions

Physiologically, sutures usually experience extrinsic impact loading such as headbutting in goats and beak-pecking in woodpeckers. However, little information is available describing the role of sutures under physiological dynamic loading conditions. In this study FE models were analyzed in three suture morphologies to obtain a better understanding of the response of bone-suture structures to impulsive loadings.

With regard to attenuation of dynamic stress in the bone-suture complex, the results shown in Figure 3 indicate that increasing the number of hierarchical orders tended to decrease the peak sectional stress in R-bone. The reason for this behavior is that the amount of transmitted stress wave from suture interface to R-bone would decrease as the increased number of hierarchies in the suture interface, based on the stress wave theory [23]. On the contrary, increasing the suture elastic modulus (*E*_*s*_) led to increasing the peak sectional stress in R-bone. This is obviously due to the increasing ratio of the acoustic impedance of suture to the acoustic impedance of bone [24]. In addition, changing the collagen fiber orientation would produce changes in the acoustic impedance of suture, resulting in the variations in the peak sectional stress. Therefore, the higher-order hierarchical suture, lower suture elastic modulus, and the better fiber orientation must have a greater potential for impact attenuation.

Results from the time histories of SUI for all isotropic and orthotropic models suggested that the suture morphologies played a major role in distributing the impact loads applied to the bone-suture structures. Due to the interface scattering effects, the hierarchical suture could make the distribution of dynamic stress within bones rapidly reach uniformity. The uniform stress indicates that most of the bone is bearing the applied load and, hence, the material is optimally used, providing a weight and a volume advantage to meet a given strength. It was noted by Li et al. [7] that the spatial homogeneity of the stress also has implications on fatigue life where suture joints often undergo cyclic tensile loading as part of their function.

However, it was found in Figure 5(a) that there were greater stress areas marked by the black arrows in the L-bone of model B, compared with model A and model C. Correspondingly, the stress values were seen in the zone marked by the arrow, as shown in Figure 5(b). From a stress wave point of view, the greater stress values were caused by the superposition of the reflected elastic wave at the interface between the L-bone and the suture. To avoid this, the actual suture usually forms a complex interdigitated structure that has a noninteger fractal dimension [25].

**Figure 10:**
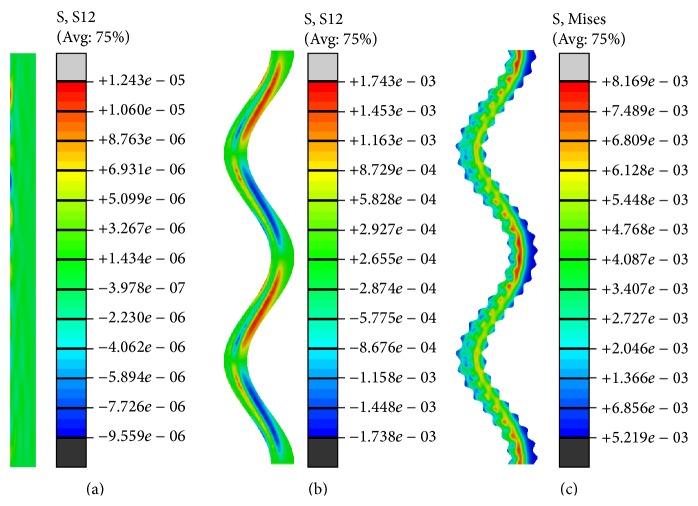
von Mises stress fields in different suture morphologies at 4 *μ*s: (a) straight suture, (b) pure sinusoidal suture, and (c) two-order hierarchical sinusoidal suture.

In both isotropic models and orthotropic models under impulsive loading, the suture strain energy ratio e increased as the number of hierarchies of the suture morphology increased, regardless of the suture elastic modulus and the collagen fiber orientation. It is indicated that the hierarchical suture was the best suture morphology for storing energy, which was consistent with previous hypotheses that interdigitated sutures were optimized for withstanding high compressive strain [26] and could absorb more energy [3]. Intrinsically, the interdigitating hierarchical suture would create additional load resistance capability to raise the performance in storing strain energy. For example, the shear stress in the interdigitating hierarchical suture was much greater than the one in the straight suture (Figure 10).

In the orthotropic models, variations in the suture strain energy ratio with changing collagen fiber orientation indicate that an optimal fiber arrangement would make the suture better to withstand both tension-compression loading and shear loading, imparting a greater potential for storing energy. This would be inconsistent with the findings of the interdigitated bone suture under a static loading [14].

Importantly, it should be noted that the finite element models used in this analysis are limited in certain respects. First, they were simplified structurally to reduce model complexity and avoid the three-dimensional effects of the real bone-suture structure. In reality, the real sutures can exhibit highly variable degrees of interdigitation and are believed to change the dynamic stress distributions resulting from flexural wave in the suture and surrounding bone. In addition, we purposely excluded other stress attenuation mechanisms such as the viscoelastic property of the suture and bony connectivity in order to evaluate the stress attenuation contribution by the suture morphologies and suture material properties (Young's modulus and collagen fibre orientation). Nonetheless, the models developed in this study provide a useful framework for understanding the underlying fundamental stress attenuation mechanisms of bone-suture structures. Also, the models may facilitate a better understanding of the evolution of biological sutures.

## 5. Conclusions

The finite element models developed in this study highlighted the mechanical behavior of bone-suture structures under impulsive loadings. With reference to suture anisotropy and morphology, the roles of suture elastic modulus and collagen fiber orientation on stress attenuation, distribution of dynamic stress within bone, and suture strain energy were quantitatively explored.

The key finding of this study was that the morphology of the suture had large influences on the dynamics of the bone-suture complex. With the increased number of hierarchical orders, the dynamic strain energy of the suture increased in the isotropic models and the orthotropic models. The higher-order hierarchical suture, serving as a high effective transmission barrier, could be better to attenuate the dynamic stress in the bone-suture complex. Furthermore, the hierarchical suture could efficiently make the distribution of dynamic stress within bones more homogeneous, compared with the straight suture. The suture elastic modulus and the arrangement of sutural fibers also had influence on the dynamics. Our simulations revealed that both the suture with high elasticity and appropriate fiber orientation benefited the stress attenuation and energy absorption.

Several questions remain. As we know, the actual bone-suture complexes usually are shell structures. How does the curvature of the stiff bone affect the dynamic behavior of the bone-suture complex during impact loading? In addition, the effects of viscoelastic suture on the bone-suture complex subjected to impulsive loadings are not considered here. For further modeling, experiments and comparative anatomy may answer such questions.
